# Prognostic value of long noncoding RNA urothelial carcinoma-associated 1 in esophageal carcinoma: A protocol for meta-analysis, TCGA data and bioinformatics analysis

**DOI:** 10.1097/MD.0000000000025452

**Published:** 2021-04-23

**Authors:** Hong Zhang, Jie Tian, Jianming Tang, TianHu Wang

**Affiliations:** a Department of Thoracic Surgery, The Third Affiliated Hospital of Chongqing Medical University, Chongqing, 401120, China.

**Keywords:** esophageal cancer, long noncoding RNAs, meta-analysis, prognosis, urothelial carcinoma associated 1

## Abstract

**Background:**

Currently, an increasing number of long noncoding RNAs (LncRNAs) have been reported to be abnormally expressed in human carcinomas and play a vital role in tumourigenesis. Some studies have been carried out to investigate the influence of the expression of LncRNA human urothelial carcinoma associated 1 (UCA1) on prognosis and clinical significance in patients with esophageal cancer, but the results are contradictory and uncertain. A meta-analysis and was conducted with controversial data to accurately assess the issue. We collected relevant TCGA data to further testify the result. In addition, bioinformatics analysis was conducted to investigate the mechanism and related pathways of LncRNA UCA1 in esophageal carcinoma.

**Methods:**

Wanfang, Chinese Biomedical Literature Database, Chinese National Knowledge Infrastructure, the Chongqing VIP Chinese Science and Technology Periodical Database, PubMed, Embase, and Web of Science were thoroughly searched for relevant information. Two reviewers independently performed data extraction and literature quality evaluation. Odd ratio and its 95% confidence intervals were applied to evaluate the relationship between LncRNA UCA1 and clinicopathological characteristics of esophageal carcinoma patients. Hazard ratios and its 95% confidence intervals were adopted to assess the prognostic effects of LncRNA UCA1 on overall survival and disease-free survival. Meta-analysis was performed with Stata 14.0 software. To further assess the function of LncRNA UCA1 in esophageal carcinoma, relevant data from The Cancer Genome Atlas (TCGA) database was collected. Three databases, miRWalk, TargetScan, and miRDB, were used for prediction of target genes. Genes present in these 3 databases were considered as predicted target genes of LncRNA UCA1. Venny 2.1 were used for intersection analysis. Subsequently, GO, KEGG, and PPI network analysis were conducted based on the overlapping target genes of LncRNA UCA1 to explore the possible molecular mechanism in esophageal carcinoma.

**Results:**

This study provides a high-quality medical evidence for the correlation between LncRNA UCA1 expression and overall survival, and between disease-free survival and clinicopathological features. Based on bioinformatics analysis, this study enhanced the understanding of the mechanism and related pathways of LncRNA UCA1 in esophageal carcinoma.

**Conclusion:**

The study provides updated evidence to evaluate whether the expression of LncRNA UCA1 is in association with poor prognosis in patients with esophageal carcinoma.

**Ethics and dissemination:**

The private information from individuals will not be published. This systematic review also should not damage participants’ rights. Ethical approval is not available. The results may be published in a peer-reviewed journal or disseminated in relevant conferences.

**OSF Registration Number:**

DOI 10.17605/OSF.IO/8MCHW.

## 1 Introduction

The incidence of esophageal carcinoma ranks eighth among all malignant tumors, and the fatality rate is at the sixth place among all malignant tumors.^[1]^ The pathological types of esophageal carcinoma mainly include squamous cell carcinoma and adenocarcinoma. About 90% of esophageal cancer in China is squamous cell carcinoma.^[2]^ At present, the prognosis of patients with esophageal carcinoma is mainly judged by clinical stage and pathological grade, but patients with the same clinicopathological features may manifest different prognosis.^[3]^ Therefore, it is particularly important to find potential prognostic biomarkers of esophageal carcinoma.

The incidence of esophageal carcinoma is caused by many factors. With the development of high-throughput gene sequencing technology, long-stranded non-coding RNA (LncRNA) is considered to be important in the development of many kinds of cancers.^[4–7]^ Recent evidence suggests that LncRNA is abnormally regulated in a variety of human cancers.^[8–11]^ LncRNA participates in the proliferation, migration and invasion of tumor cells through various mechanisms.^[12–15]^ In addition, the overexpression of LncRNA is associated with the clinicopathological features of various cancers with high levels of LncRNA, which indicates poor overall survival (OS) and disease-free survival (DFS).^[16,17]^ Therefore, more and more attentions have been paid to the clinical application of LncRNA that is regarded as a potential biomarker and therapeutic target for a lot of cancers.

Human urothelial carcinoma associated 1 (UCA1) refers to cancer-regulated drug resistance gene that involves in the development of different malignant tumors, such as gastric cancer, lung cancer, rectal cancer and so on.^[18–21]^ Derived from the family of human endogenous retroviruses, LncRNA UCA1 is LncRNA molecule. Its gene is located on human chromosome 19 and its full length is 1.4 kb. LncRNA UCA1 was first discovered in bladder cancer and can promote tumor cell proliferation and metastasis.^[22]^

A number of studies have revealed that the high expression of LncRNA UCA1 is closely related to the survival of patients with esophageal carcinoma, but the results are also quite different.^[23–28]^ To explore the expression and potential biological processes of LncRNA UCA1 in esophageal carcinoma, we conducted meta-analysis to explore the role of LncRNA UCA1 in esophageal carcinoma, and collected relevant The Cancer Genome Atlas (TCGA) data to further testify the result. In addition, bioinformatics analyses including Gene ontology (GO), Kyoto Encyclopedia of Genes and Genomes (KEGG) and the protein-protein interaction (PPI) network analysis were conducted based on the overlapping target genes of LncRNA UCA1 to explore the possible molecular mechanism in esophageal carcinoma.

## 2 Methods

### 2.1 Study registration

The protocol of the systematic review has been registered on Open Science Framework. The registration number is DOI 10.17605/OSF.IO/8MCHW. This meta-analysis protocol is based on the Preferred Reporting Items for Systematic Reviews and Meta-analysis Protocols (PRISMA-P) Statement Guidelines.^[29]^

### 2.2 Data sources and search strategy

Wanfang, Chinese Biomedical Literature Database, Chinese National Knowledge Infrastructure, the Chongqing VIP Chinese Science and Technology Periodical Database, PubMed, Embase, and Web of Science are our electronic databases for retrieval. The retrieval time is from their inception to January 2021. The retrieval strategy will be created by researchers’ discussions on the basis of the Cochrane handbook guidelines. The search strategy for PubMed is illustrated in Table 1. According to the actual situation of other electronic databases, the retrieval strategy can be modified.

**Table 1:** Search strategy in PubMed database.

Number	Search terms
#1	Esophageal Neoplasms[MeSH]
#2	Cancer of Esophagus[Title/Abstract]
#3	Esophageal Cancer[Title/Abstract]
#4	Cancer of the Esophagus[Title/Abstract]
#5	Esophagus Cancer[Title/Abstract]
#6	Esophagus Neoplasm[Title/Abstract]
#7	Neoplasms, Esophageal[Title/Abstract]
#8	Cancer, Esophageal[Title/Abstract]
#9	Cancer, Esophagus[Title/Abstract]
#10	Cancers, Esophageal[Title/Abstract]
#11	Cancers, Esophagus[Title/Abstract]
#12	Esophageal Cancers[Title/Abstract]
#13	Esophageal Neoplasm[Title/Abstract]
#14	Esophagus Cancers[Title/Abstract]
#15	Esophagus Neoplasms[Title/Abstract]
#16	Neoplasm, Esophageal[Title/Abstract]
#17	Neoplasm, Esophagus[Title/Abstract]
#18	Neoplasms, Esophagus[Title/Abstract]
#19	or/1-18
#20	UCA1[Title/Abstract]
#21	Human urothelial carcinoma associated 1[Title/Abstract]
#22	or/20-21
#23	prognos∗[Title/Abstract]
#24	survival[Title/Abstract]
#25	or/23-24
#26	#19 and #22 and #25

### 2.3 Inclusion criteria for study selection

The included articles must meet the following inclusion criteria:

(1) Patients who were diagnosed with esophageal carcinoma based on pathology and histology.
(2) The expression of LncRNA UCA1 in related esophageal carcinoma tissues.
(3) Reported LncRNA UCA1 survival-related data, including OS and DFS.
(4) Patients were divided into LncRNA UCA1 positive (high) and LncRNA UCA1 negative (low).
(5) The relationship between clinicopathological parameters and prognosis of LncRNA UCA1 was introduced in details.
(6) Published as full-text articles, original Chinese and English Research papers.

The criteria for excluding literatures are summarized as follows:

(1) Relevant critical articles or conference articles and related case reports or replying emails.
(2) Documents that cannot be tested in electronic database.
(3) Non-human experiments were carried out.
(4) It was impossible to accurately express LncRNA UCA1 and count the clinical characteristics and overall survival data of breast cancer.
(5) Repeatedly published literature.

### 2.4 Data collection and analysis

#### 2.4.1 Selection of studies

**Figure 1:**
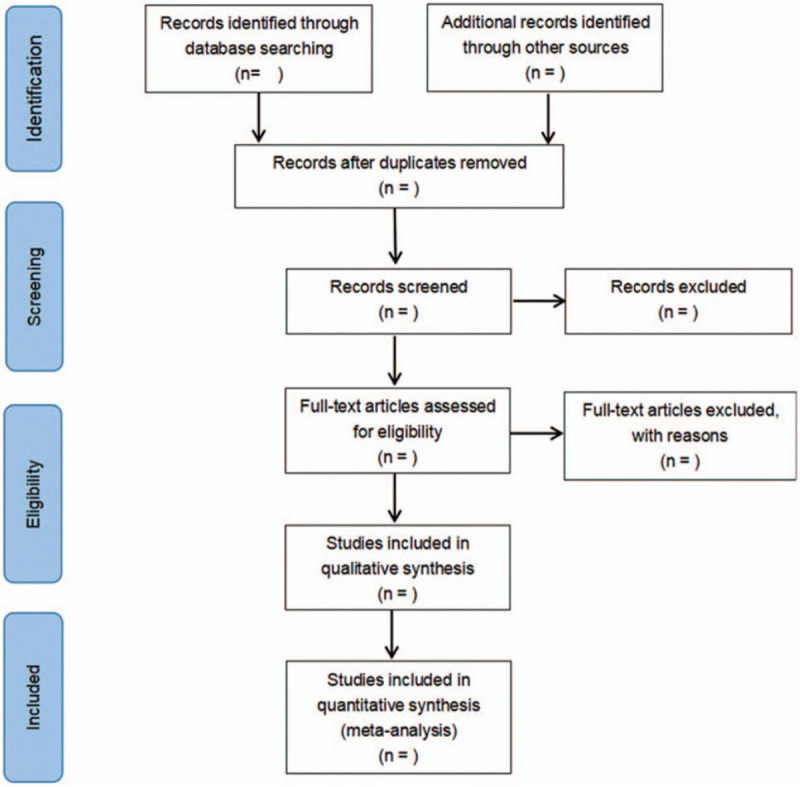
Flow diagram of study selection process.

The two reviewers independently screened literatures according to the title, abstract and key words, and excluded irrelevant literatures. The rest of literatures will be further confirmed by two researchers by reading the full text. The excluded researches and the reasons for the exclusion will be recorded. The differences between the two reviewers will be resolved through consensus or a third independent reviewer. The literature screening process is demonstrated in Figure 1.

#### 2.4.2 Data extraction and management

According to the inclusion and exclusion criteria, two reviewers independently extracted data from the eligible studies. Data were double input into EpiData software (version 3.0; The EpiData Association, Odense, Denmark) by two reviewers. Differences were resolved through group discussion. The information extracted includes the first author name, publication year, ethnicity, total patient number, detection method for LncRNA UCA1 expression, number of patients in high and low LncRNA UCA1 expression groups, survival analysis method, cut-off value to define the high and low LncRNA UCA1 expression, HRs and corresponding 95% CIs for OS, and DFS, if provided. The data were analyzed by both univariate and multivariate methods, and the latter was preferred. We obtained hazard ratios (HRs) and confidence intervals (95%CIs) from the Kaplan-Meier survival curves with Engauge Digitizer version 4.1 (http://digitizer.sourceforge.net/).

### 2.5 Assessment of quality in included studies

The quality of all the included studies will be evaluated by two reviewers independently according to the Newcastle-Ottawa scale (NOS) that is used to evaluate the quality of observational studies.^[30]^ The NOS values arrange from 0 to 9. Studies with the score of 6 are considered to be of high quality.^[31]^

### 2.6 Measures of prognosis

OS and DFS will be taken as prognostic outcomes. The results will be expressed as HRs, with 95% CIs.

### 2.7 Management of missing data

If there is insufficient or missing data in the literature, we will contact the author via email. If the data is not available, we will only analyze the current available data and discuss its potential impacts.

### 2.8 Statistical analysis

Statistical analysis was performed with STATA 14.0 (STATA Corporation, College Station, TX). HRs and 95% CIs was used to evaluate the inter-relationship among LncRNA UCA1 expression and OS and DFS. Odds ratio and 95% CIs were applied to evaluate the impacts of LncRNA UCA1 expression on clinicopathological characteristics. First, statistical heterogeneity tests were performed on the included studies. If there is no statistical heterogeneity among the included literatures (I2<50%, P≥0.1), a fixed effect model will be adopted. When there is statistical heterogeneity among the included literatures (P< 0.1, I2 > 50%), the sources of heterogeneity will be analyzed. Clinical heterogeneity will be treated by subgroup analysis. In the absence of significant clinical heterogeneity and methodological heterogeneity, statistical heterogeneity will be considered, and random effects models will be adopted for analysis. If the clinical heterogeneity of the subgroup analysis is significantly higher, no meta-analysis will be performed, with only a descriptive analysis.

### 2.9 Additional analysis

#### 2.9.1 Subgroup analysis

We will conduct a subgroup analysis based on the detection method of LncRNA-UCA1expression, race, and the sources of survival data.

#### 2.9.2 Sensitivity analysis

The sensitivity analysis of each index was carried out through elimination method to check the stability of the results.

#### 2.9.3 Reporting bias

If the number of studies included in a certain outcome index is no less than 10, funnel chart will be applied to evaluate publication bias.^[32,33]^

### 2.10 TCGA data collection

To further assess the function of LncRNA-UCA1 in esophageal carcinoma, relevant data from the TCGA database (https://cancergenome.nih.gov/) was collected. Overall survival (OS), disease-free survival were all analyzed by univariate Cox regression, and the relation between LncRNA-UCA1 and the clinicopathological parameters was assessed by the Independent-sample t test. The statistical analysis was conducted on SPSS statistical software package, version 21.0 (IBM Corporation, Armonk, NY), and P < .05 indicates significant.

### 2.11 Bioinformatic analysis

Three databases, miRWalk, TargetScan, and miRDB, were used for prediction of target genes. Genes present in these 3 databases were considered as predicted target genes of LncRNA-UCA1. Online tool, Venny 2.1 (https://bioinfogp.cnb.csic.es/tools/venny/), were used for intersection analysis. Subsequently, GO, KEGG, and PPI network analysis were conducted based on the overlapping target genes of LncRNA-UCA1 to explore the possible molecular mechanism in osteosarcoma. GO can describe the functions of gene products from all organisms.GO annotation includes 3 categories: biological process, cellular component and molecular function. KEGG is a comprehensive knowledge base for both functional interpretation and practical application of genomic information. In the present study, GO and KEGG analyses were conducted by the clusterProfiler package of R software.P value < .05 and Q values < 0.05 were set as the cutoff criterion.Additionally, the PPI network was analyzed by the search tool for the retrieval of interacting genes database (https://string-db.org/), and target genes with target score≥80 were used for PPI analysis. The cut-off criteria were a combined score of > 0.9 for a PPI network. Disconnected nodes were hided in the network. Line thickness indicates the strength of data support. Furthermore, we used the plug-in of molecular complex detection (MCODE) app in Cytoscape 3.7.0 software to extract hub genes from the PPI network. The advanced options set as degree cutoff = 2, K-Core = 2, and Node Score Cutoff = 0.2.

### 2.12 Ethics and dissemination

The content of this article does not involve moral approval or ethical review and would be presented in print or at relevant conferences.

## 3 Discussion

In recent years, more and more studies have confirmed that lncRNA participates in the process of tumor cell proliferation, apoptosis, invasion and metastasis, and plays an important role in the occurrence and development of tumor.^[34]^ Tumorigenesis is caused by the activation of proto-oncogenes, the inactivation of tumor suppressor genes and the immortalization of cells. Normal cells have a perfect regulatory system to keep LncRNA in a dynamic balance, but this balance is broken in tumor cells.

Previous studies have proved that LncRNA UCA1 may play an important oncogene role in human cancers, including hepatocellular carcinoma, breast cancer, colorectal cancer and gastric cancer.^[8–11]^ Li et al^[23]^ revealed that the expression of LncRNA UCA1 is up-regulated in esophageal cancer tissues and cell lines, and is closely related to tumor differentiation, clinical stage and lymph node metastasis. Wang et al^[35]^ found that LncRNA UCA1 plays the role of tumor suppressor gene in esophageal carcinoma. LncRNA UCA1 decreases in patients suffering from esophageal carcinoma. Meanwhile, the increase of LncRNA UCA1 may inhibit cell proliferation, migration and invasion. Jiao et al^[24]^ reported that LncRNA UCA1 was significantly overexpressed in esophageal carcinoma tissues, compared with adjacent non-tumor tissues, and its expression was consistent with tumor stage, tumor differentiation, and predicted poor prognosis. In this study, systematic evaluation and meta-analysis were conducted to further evaluate the correlation between the expression of LncRNA UCA1 and the prognosis of patients with esophageal carcinoma.

In conclusion, our meta-analysis could provide latest evidence for the correlation between LncRNA UCA1 and the prognosis of patients suffering from esophageal carcinoma. Therefore, LncRNA UCA1 may become a potential biomarker of prognosis in patients with esophageal carcinoma. Based on bioinformatics analysis, this study enhanced the understanding of the mechanism and related pathways of LncRNA UCA1 in esophageal carcinoma.

## Author contributions

**Conceptualization:** Hong Zhang, TianHu Wang.

**Data curation:** Hong Zhang, Jie Tian.

**Formal analysis:** Jie Tian.

**Funding acquisition:** TianHu Wang.

**Methodology:** Jie Tian.

**Project administration:** TianHu Wang.

**Resources:** Jie Tian.

**Software:** Jianming Tang.

**Supervision:** Jianming Tang.

**Validation:** Jianming Tang.

**Visualization:** Jianming Tang.

**Writing – original draft:** Hong Zhang, TianHu Wang.

**Writing – review & editing:** Hong Zhang, TianHu Wang.
